# Impact of germline MMR gene variants on immune checkpoint inhibitors response in patients with MSI-H/dMMR digestive cancers: a retrospective cohort analysis

**DOI:** 10.1186/s13053-026-00339-w

**Published:** 2026-04-18

**Authors:** Antoine Dardenne, Camille Loisel, Anna Pellat, Alexandre Perrier, Julie Metras, Thomas Samaille, Yann Parc, Julie Leclerc, Romain Cohen, Thierry André

**Affiliations:** 1 Department of Medical Oncology, Saint-Antoine Hospital, AP-HP , Sorbonne University, Paris, 75012 France; 2 Department of Genetics, AP-HP, Pitié-Salpêtrière University Hospital, Sorbonne University, Paris, France; 3 Department of Digestive Surgery, AP-HP, Saint-Antoine Hospital, Sorbonne University, Paris, France; 4 Digestive Oncology Department, Gastroenterology, Digestive Endoscopy, Cochin Hospital, AP-HP Centre, Paris, France; 5 Faculty of Médecine, Paris Cité University, Paris, France; 6 Paris Cité University and Sorbonne Paris Nord University, INSERM, INRAe, Center for Research in Epidemiology and Statistics (CRESS), Hôtel Dieu Hospital, Paris, France; 7 Lille University, CNRS, INSERM, CHU Lille, UMR9020-U1277 - CANTHER - Cancer Heterogeneity Plasticity and Resistance to Therapies, Lille, France; 8 Molecular Oncogenetics Unit, Department of Biochemistry and Molecular Biology, Lille University Hospital, Lille, France; 9 Unité Mixte de Recherche Scientifique 938 and SIRIC CURAMUS, Centre de Recherche Saint-Antoine Hospital, Equipe Instabilité des Microsatellites et Cancer, Equipe Labellisée par la Ligue Nationale Contre le Cancer, Inserm, Paris, 75012 France

**Keywords:** Lynch syndrome, MSI-H/dMMR, Digestive cancers, Immune checkpoint inhibitors, Germline MMR variants, Immunotherapy response

## Abstract

Immune checkpoint inhibitors (ICIs) have transformed outcomes in microsatellite instability-high (MSI-H) deficient mismatch repair (dMMR) cancers. The influence of germline MMR gene variants on ICI remains unclear. In this single-center study, 93 patients with Lynch syndrome-associated MSI-H/dMMR digestive cancers received ICI monotherapy or combination therapy. Germline MMR variants were classified, and progression-free survival (PFS) was evaluated by gene and variant type. Most patients carried *MLH1* or *MSH2* variants. At 24 months, estimated PFS was 81.5% for *MLH1*, 67.2% for *MSH2/EPCAM*, and 78.6% for *MSH6*, with no PFS events in *MLH1* promoter methylation or *PMS2* subgroups. No statistically significant differences in PFS were observed between gene groups. Performance status ≥ 2 was the only factor associated with poorer PFS. Neither MMR gene type nor variant class significantly influenced ICI efficacy in Lynch syndrome-related MSI-H/dMMR digestive cancers, supporting the use of ICIs regardless of germline MMR genotype.

## Introduction

Immune checkpoint inhibitors (ICIs) have substantially improved clinical outcomes of patients with microsatellite instability-high (MSI-H)/deficient mismatch repair (dMMR) cancers [1, 2]. Nevertheless, a proportion of patients experience primary resistance, and others develop acquired resistance despite an initial response [3]. This variability underscores a critical need for predictive biomarkers to optimize treatment selection while balancing efficacy and toxicity. Several candidate markers have been investigated. Notably, PD-L1 expression and *BRAF* mutations have not been associated with improved ICI response. Although tumor mutational burden (TMB) has shown consistent predictive value in some studies, diagnostic variability, particularly in immunohistochemistry assessment, may affect its reliability [4]. Omics-based signatures are promising [5, 6] but require further validation. Moreover, germline genetic data remain limited or incomplete in most studies evaluating ICIs.

Interestingly, MSI-H tumors arising in patients carrying a germline pathogenic variant in one of the MMR genes may be more responsive to ICIs than sporadic MSI-H tumors [3, 7, 8]. Furthermore, both the type of gene alteration and the mechanism of gene inactivation (mutation versus promoter hypermethylation) have been shown to impact overall survival (OS) in patients treated with ICIs [7]. However, data on gene-specific effects on immunotherapy response remain limited. This study aimed to evaluate ICI efficacy according to MMR gene and variant type in Lynch syndrome-associated MSI-H/dMMR digestive cancers.

## Methods

### Study design and cohort

This study is a retrospective analysis of a prospective single-center cohort from Saint-Antoine Hospital, Paris. The study population included all patients with MSI-H/dMMR digestive cancers treated with an anti- Programmed cell Death protein-1 (PD1) monotherapy or combined anti-PD-1 and anti-CTLA-4 ICI at Saint-Antoine Hospital between February 2015 and April 2024. MSI-H/dMMR status was determined locally using immunohistochemistry and multiplex polymerase chain reaction. A total of 260 patients were enrolled in the ImmunoMSI cohort. After excluding 167 patients without confirmed germline pathogenic variant, 93 patients with molecularly confirmed Lynch syndrome were included in the final analysis.

### Data collection and ethical considerations

Data were extracted from the ImmunoMSI database and from patients’ medical records. Pathogenic and likely pathogenic variants were classified into four categories by a specialist in molecular and medical genetics at Pitié Salpêtrière Hospital (APHP): missense/indel phase, splicing variants, large genomic rearrangement, and truncating variants (nonsense and frameshift). Variant interpretation followed the American College of Medical Genetics and Genomics (ACMG) guidelines. The database was approved by the ethics committee (CER-2024-AMARA-ImmunoMSI - MS1.0-00267). Non-objection for the use of routine care data and database information was obtained from all patients, except those deceased. The cohort study was approved by the ethics committee (N°2020 – CER 2020-6).

### Statistical analysis and definitions

Data analysis was performed using Rstudio software (version 1.2.5033). Categorical variables were summarized as counts and percentages, and continuous variables as means with standard deviations or medians with interquartile ranges, along with their range. Progression-free survival (PFS) was defined as the time between the start of treatment with ICIs and the occurrence of disease progression or death. Radiologic response was assessed by expert radiologists according to Response Evaluation Criteria in Solid Tumors (RECIST) 1.1 and immune (iRECIST). Survival analyses were performed using the Kaplan-Meier method. Associations between predefined variables and PFS were evaluated using Cox proportional hazards models. The following parameters were assessed for their impact on ICI response: age, sex, liver and peritoneal metastases, performance status (PS; PS 0/1 versus PS ≥ 2) at treatment initiation, type of ICI regimen (monotherapy versus dual blockade), the presence of mutations (*MLH1*,* MSH2/EPCAM*,* MSH6*,* PMS2*,* or* constitutional *MLH1* epimutation), type of germline alteration (sequence variants: missense/indel, splicing variants, large rearrangement, truncating variants; and constitutional *MLH1* promoter hypermethylation), and treatment setting (first-line treatment versus chemotherapy with or without targeted therapy). A two-sided p value < 0.05 was considered statistically significant.

### Outcome measure

The objective of this study was to evaluate 2-year PFS following ICIs treatment in patients with Lynch syndrome-associated MSI-H/dMMR digestive cancers, according to the affected MMR gene (*MLH1*,* MSH2/EPCAM*,* MSH6*,* or PMS2)* and the type of germline variant type.

## Results

The final study population included 93 patients with MSI-H/dMMR metastatic digestive cancers. Most patients were male (67%) and had colorectal cancer (83%). Nearly half carried a pathogenic variant in *MSH2* (47%; Table 1). Fifty-two patients received anti-PD-1 or anti-PD-L1 monotherapy, and 41 received combined anti-PD-1 and anti-CTLA-4 therapy. The median follow-up from ICI initiation to last contact or death was 47.8 months.

**Table 1:** Baseline characteristics of patients with Lynch syndrome–associated MSI-H/dMMR digestive cancers (*N* = 93)

Median age		*N* = 93	*N*/93 (%)
		62	-
**Sex**			
Male		62	67
Female		31	33
**Primary tumor**			
Colorectal		78	83
Pancreas		4	4
Stomach		4	4
Small intestine		6	6
Bile ducts		1	1
**Gene**			
*MLH1*		28	30
*MSH2/EPCAM*		44	47
*MSH6*		14	15
*PMS2*		4	4
*MLH1* Promoter Methylation		3	4
**Personal history of Lynch spectrum cancer prior ** **to ICIs treated metastatic cancer**		39	42
**Epigenetic mechanism**			
*MLH1* promoter methylation	3		3
**Type of pathogenic variant**			
Missense		19	21
Splicing		20	22
Large rearrangement		14	15
Truncating		30	34
Missing data		7	8
**MMR status**			
pMMR		2	2
dMMR		91	98
**MSI status**			
MSI High		71	76
Missing data		22	24

According to iRECIST criteria, most patients achieved either a partial response (41%) or a complete response (32%). Stable disease was observed in 12% and primary progressive disease in 15%. Among the 28 patients who progressed on ICIs, 7 of 11 patients with stable disease later progressed (63.6%), and 7 of 38 patients with a partial response progressed (18.4%). Notably, no progression event occurred among the 30 patients who achieved a complete response.

**Fig. 1:**
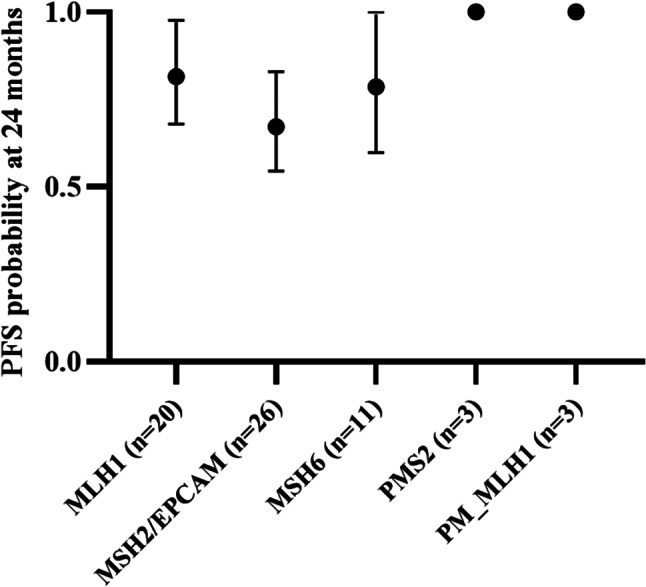
Estimated 2-year progression-free survival by MMR gene group. Bars represent the 24-month PFS for patients with MLH1, MSH2/EPCAM, MSH6, PMS2, and MLH1 promoter methylation (PM_MLH1). Error bars indicate 95% confidence intervals

At 24 months, the estimated PFS was 81.5% (95% CI 68.0–97.6) for *MLH1*, 67.2% (95% CI 54.5–82.9) for *MSH2/EPCAM*, and 78.6% (95% CI 59.8–100) for *MSH6*. No PFS events were observed in the *MLH1* promoter methylation or *PMS2* subgroups within the first two years of follow-up (Fig. 1). Thirty patients were censored at data cutoff, including 28 who progressed after 24 months and two who had not yet reached 24-month follow-up. Objective responses were observed across tumor sites, with no apparent signal of differential efficacy, although subgroup sizes were small.

Using *MLH1* as the reference group, there was no statistically significant difference in PFS according to the affected gene (*MSH2/EPCAM*: *p* = 0.1; *MSH6*: *p* = 0.7), and no PFS events occurred in the *MLH1* promoter methylation or *PMS2* groups (Fig. 1). Multivariate analysis did not reveal any significant differences in PFS, which was not reached in any subgroup (Table 2). Two tumors were pMMR by IHC but MSI by pentaplex, both harboring a pathogenic missense germline *MSH2* variant and responding to ICI.

**Table 2:** Univariate and multivariate analysis of factors associated with progression-free survival

	Univariate analysis		MULTIVARIATE ANALYSIS	*p*-value
	HR (IC 95%)	*p*	HR (IC 95%)	
**Type of gene (Ref = ** ***MLH1)***				
*MSH2/EPCAM* *MSH6* *PMS2* *MLH1* promoter methylation	2.1 (0.8–5.3) 1.3 (0.4–4.5) 1.2 (0.1–10.3) < 0.1	0.1 0,7 0,8 1	3.0 (0.7–12.0) 2.2 (0.4–11.6) 0.3 (0.01–8.4) NA	0.1 0.4 0.5 NA
**Age** (Ref: < 50 years)				
50-70> 70 years	1.7 (0.7–3.9) 2.6 (0.8-8.0)	0.3 0,09	2.6 (0.9–7.6) 2.6 (0.5–14.0)	0.09 0.3
**Liver metastasis**	0.9 (0.5-2.0)	0.9	1.6 (0.6–4.7)	0.4
**Peritoneal carcinosis**	0.8 (0.4–1.7)	0.5	1.0 (0.3-3.0)	1
**PS ≥ 2**	5.7 (2.0-16.8)	0.001*	4.5 (0.7–31.2)	0.10
**Male**	1.4 (0.6–3.3)	0.4	1.8 (0.6–5.5)	0.319
**≥ second-line treatment**	1.5 (0.6–3.9)	0.4	2.9 (0.8–10.5)	0.1
**Type of ICI** (Ref: Combination therapy) Monotherapy	2.0 (0.9–4.4)	0.09	2.1 (0.8–5.3)	0.1
Type of mutation (Ref: Missense)				
Splicing Large rearrangement Truncating	0.4 (0.1–1.5) 1.5 (0.5–4.5) 0.6 (0.2–1.7)	0.2 0.5 0.4	0.4 (0.08–1.6) 1.7 (0.4–7.7) 0.4 (0.1–1.6)	0.2 0.5 0.2

Only PS ≥ 2 was associated with worse PFS in univariate analysis (HR 5.7, 95% CI 2.0–16.8, *p* = 0.001), although this association did not remain significant in multivariate analysis (HR 4.5, 95% CI 0.7–31.2, *p* = 0.10).

## Discussion

In our series, patients with constitutional *MLH1* epimutation, *MSH6*, or *PMS2* variants demonstrated response rates comparable to those observed in other Lynch syndrome carriers. Previous studies showed that loss of *MSH6*, through disruption of the MutSα complex, may lead to the development of less immunogenic tumors [9], characterized by a distinct mutational profile enriched in single-nucleotide variants. Other reports have described reduced immune cell infiltration in tumors with isolated PMS2 loss [10], and lower TMB [11], suggesting that immunogenicity may vary depending on the affected MMR gene [12].

The strengths of this study include its long median follow-up, expert validation of all germline variant, and size of the cohort, which represents the largest known series reported to date. Limitations include the heterogeneity of tumor types and treatment regimens. In addition, variant classification was not available for seven patients followed in external genetic laboratories; although germline pathogenicity was confirmed clinically, detailed variant nomenclature could not be retrieved.

In a recent poster presentation, Randrian et al. [7] showed that tumors with *MLH1* epimutation or isolated loss of *PMS2* were associated with shorter OS in two large pan-cancer cohorts (MSKCC, *n* = 1,958 and Caris, *n* = 13,421). However, isolated *PMS2* loss in immunohistochemistry may result from heterogeneous or undetected *MLH1* promoter hypermethylation rather than a true Lynch syndrome-related defect [13]. The authors also highlighted that Lynch syndrome remains the major prognostic factor associated with MSI-H status, regardless of the affected MMR gene. These findings suggest that while the mechanism of MMR inactivation may influence ICIs response, their specific impact in the germline setting requires dedicated analyses such as the presented study.

External validity is supported by the consistency of our findings with data from a Chinese cohort [14], and additional data presented by Randrian et al. at the 2025 American Society of Clinical Oncology meeting [15], neither of which demonstrated significant differences in ICIs response according to the affected gene (*MLH1*, *MSH2*, or *MSH6*). In the Dana-Farber series, PFS among *PMS2*-associated MSI-H tumors was 41 months, compared with not reached for other Lynch-associated MMR variants; however, the PMS2 subgroup included only six patients. In the Chinese cohort, none of the three *PMS2* carriers achieved a partial or complete response, limiting interpretation.

These findings are consistent with recent data reported by Randrian et al. [7] indicating that PMS2-only and MSH6-only tumors are less frequently MSI-H, particularly in cancer types not typically associated with Lynch syndrome. However, next-generation sequencing has shown limited sensitivity for MSI detection in extra-colorectal and extra-endometrial tumors, with 310 dMMR cases misclassified as MSS among 1.995 tumors [16]. These limitations underline the need for more sensitive and accurate molecular tools, such as MSI Sensor, which shows performance comparable to the pentaplex assay [17], particularly for MSH6-deficient tumors [18].

## Conclusion

Currently, neither the specific MMR gene involved nor the type of germline variant appears to predict differential response to ICIs, and these factors cannot be used to guide the choice between monotherapy and combination therapy.

## Data Availability

These data are available upon reasonable request to the authors.
